# Lignin-Inspired Polymers with High Glass Transition Temperature and Solvent Resistance from 4-Hydroxybenzonitrile, Vanillonitrile, and Syringonitrile Methacrylates

**DOI:** 10.1021/acssuschemeng.1c07048

**Published:** 2021-12-07

**Authors:** Olivier Bonjour, Hannes Nederstedt, Monica V. Arcos-Hernandez, Siim Laanesoo, Lauri Vares, Patric Jannasch

**Affiliations:** † Center for Analysis and Synthesis, Department of Chemistry, Lund University, P.O. Box 124, SE-22100 Lund, Sweden; ‡ Institute of Technology, University of Tartu, Nooruse 1, Tartu 50411, Estonia

**Keywords:** Vanillin, Syringaldehyde, Lignocellulose, Biobased plastics, Copolymers, Acrylate polymers, Glass transition temperature

## Abstract

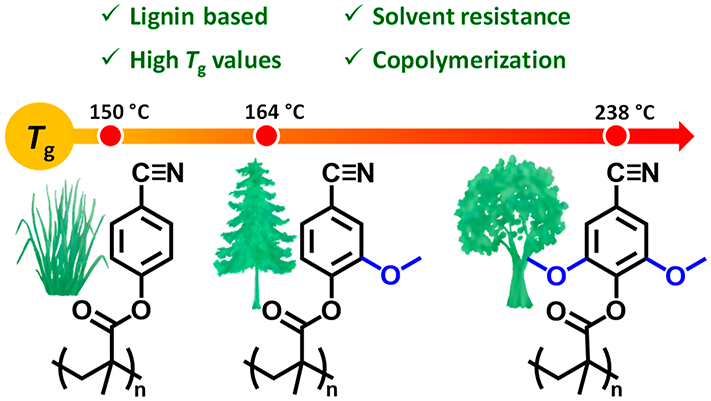

We here report on the synthesis and polymerization of nitrile-containing methacrylate monomers, prepared via straightforward nitrilation of the corresponding lignin-inspired aldehyde. The polymethacrylates reached exceptionally high glass transition temperatures (*T*_g_ values), i.e., 150, 164, and 238 °C for the 4-hydroxybenzonitrile, vanillonitrile, and syringonitrile derivatives, respectively, and were thermally stable up to above 300 °C. Copolymerizations of the nitrile monomers with styrene and methyl methacrylate, respectively, gave potentially melt processable materials with tunable *T*_g_ values and enhanced solvent resistance. The use of lignin-derived nitrile-containing monomers represents an efficient strategy toward well-defined biobased high *T*_g_ polymer materials.

## Abstract 2

Lignin-based highly polar methacrylate monomers are synthesized and polymerized to obtain green and solvent resistant polymers with high glass transition temperatures.

## Introduction

The development of new biobased thermoplastic polymers from sustainable feedstocks is essential when addressing the issues caused by fossil-based plastics.^1−6^ In order to compete with and replace fossil-based conventional plastics, biobased polymers must be produced from inexpensive and sustainable natural sources, have suitable thermal and mechanical properties, and be readily processable.^7^ This is perhaps especially difficult to achieve when it comes to amorphous thermoplastic polymers with high glass transition temperatures (*T*_g_ > 100 °C) because of the great challenge to produce this kind of material from biobased feedstocks.^8^ The *T*_g_ indicates the thermal transition between glassy (hard) and rubbery (soft) materials and is the most characteristic property of an amorphous polymer. Consequently, to a large degree, it determines the maximum use temperature and possible application areas.

**Scheme 1:**
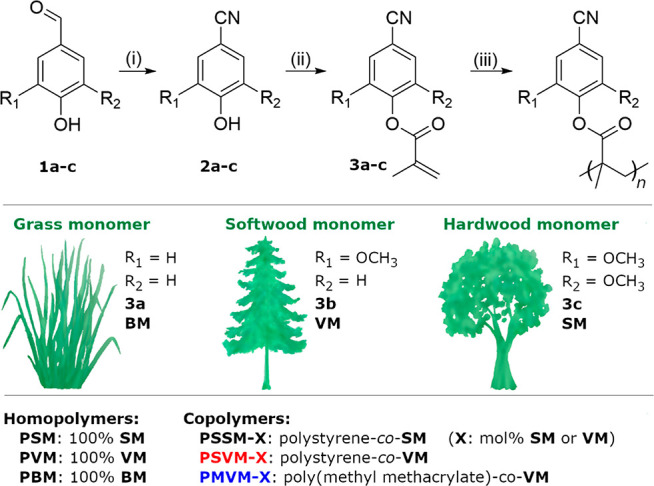
Synthetic Pathway, Nomenclature, and Molar Compositions of Lignin-Inspired Nitrile-Containing Methacrylate Monomers and Polymers Reagents and conditions: (i) hydroxylamine-*O*-sulfonic acid (1.1 equiv), acetic acid (1 equiv)/water, 50 °C, 6 h (yield 79%–82%); no column chromatography required; (ii) methacrylic anhydride (1.01 equiv), catalytic DMAP (2 mol %), ethyl acetate, 60 °C, 24 h (yield 70%–89%); (iii) AIBN (0.1–1 mol %), DMSO, 60 °C, 24 h.

The incorporation of aromatic or inflexible aliphatic ring structures to restrict the macromolecular chain mobility is a common and efficient strategy to increase the *T*_g_ and the thermal stability of polymers.^9^ Consequently, cyclic compounds like terpenes,^8,10−13^ vanillin,^14,15^ glucose,^16^ and isosorbide^8,17,18^ are common building blocks to prepare biobased high *T*_g_ polymers. The most abundant biosource of aromatic compounds is lignin, a byproduct of the paper and pulp industry. Lignins are biopolymers consisting of hydroxyphenyl, guaiacyl, and syringyl units, whose relative amounts depend on the natural source. (e.g., softwood or hardwood, Scheme 1).^19−21^ The depolymerization and purification of lignin remains however a challenge,^19,22^ thus only vanillin can nowadays be isolated from lignin in large scale (ca. 3000 tons/year).^23^ A wide variety of lignin-based polymers have been prepared and studied in the past few years.^8,14,15,24^ For example, recently it was reported that softwood lignin-based polymethacrylates based on guaiacol, 4-ethylguaiacol, creosol, and vanillin, respectively, reached *T*_g_ values ranging from 111 to 129 °C, suitable for thermoplastic elastomer and binder applications.^25^ These polymethacrylates, as well as similar ones based on phenol, syringol, and syringaldehyde, respectively, have been produced and investigated for various coating applications.^26,27^ Lignin-derived building blocks, such as eugenol,^28^ have also been employed to produce densely cross-linked thermosets with high *T*_g_ values.

A far less exploited strategy to enhance and control the thermal properties of biobased polymers is to introduce strongly polar groups. The presence of these groups will increase the *T*_g_ by increasing intermolecular interactions but will also typically increase the thermal stability and enhance the solvent resistance. The nitrile group is highly polar and has been introduced into styrenic materials to improve both mechanical properties and chemical resistance. For example, poly(styrene-*co*-acrylonitrile) (SAN) has a *T*_g_ above 100 °C, depending on the acrylonitrile content.^29^ Moreover, benzonitrile methacrylate polymers have recently been predicted by artificial neural network to possess high *T*_g_.^30^ From an industrial point of view, nitrilation is readily achieved by ammoxidation, using ammonia, oxygen, and a vanadium or molybdenum oxide catalyst.^31,32^ With the aim to develop biobased high-performance polymethacrylates with high *T*_g_ values resulting from both high macromolecular chain rigidity and polarity, we have in the present work prepared three different nitrile-containing methacrylate monomers. These monomers are based on lignin-inspired building blocks, namely, 4-hydroxybenzaldehyde **1a**, vanillin **1b**, and syringaldehyde **1c**, which correspond to hydroxyphenyl, guaiacyl, and syringyl lignin units, respectively.^20^ (Scheme 1). The phenolic rings of these building blocks are substituted with 0, 1, and 2 methoxy groups, respectively, and were selected to tune the chain rigidity. The monomers were employed in free-radical polymerizations, and the resulting homopolymers were characterized with respect to thermal properties and solvent resistance. Moreover, the nitrile-containing monomers were utilized in copolymerizations with styrene (S) and methyl methacrylate (MMA), respectively, to study their influence on the properties. The thermal stability and melt processability of the copolymers were subsequently investigated by rheology measurements.

## Results and Discussion

The monomers were synthesized in one or two steps according to Scheme 1. In step (i), the aldehyde groups of 4-hydroxybenzaldehyde and syringaldehyde first underwent nitrilation. This transformation has previously been carried out by various methods. Schuerch^33^ reported the direct nitrilation of vanillin to vanillonitrile by the Schmidt reaction, using sodium azide as the nitrogen source. Lewis acids can also be used as catalysts as reported by Nimnual et al.^34^ Recently, new chemoselective methods using triflic acid as a mediating agent have been reported.^35^ In the present case, we used a greener approach developed by Quinn et al.^36^ which involves the inexpensive hydroxylamine-*O*-sulfonic acid. This nitrogen source is less hazardous compared to azides.^37,38^ Moreover, the reaction conditions have a low environmental impact since the reaction is carried in an aqueous acetic acid solution. Thus, 4-hydroxybenzonitrile **2a** and syringonitrile **2c** were readily synthesized by this procedure with reasonably high yields, i.e., 82% and 79% for **2a** and **2c**, respectively (Scheme 1, SI). The products required no purification by chromatography, thus making this process easily scalable. Vanillonitrile **2b** was obtained directly from a commercial source. Alternatively, it is possible to produce **2b** from vanillin by green catalysis^39^ or by directly converting the methyl group of 4-methylguaiacol through ammoxidation.^31,40^ The latter is of particular interest since ammoxidation is a conventional industrial process. Moreover, various other methylated aromatic compounds can be isolated from lignocellulose, thus making ammoxidation an attractive reaction in the preparation of building blocks for high *T*_g_ biobased polymers.

In step (ii), 4-hydroxybenzonitrile **2a**, vanillonitrile **2b**, and syringonitrile **2c** were reacted with methacrylic anhydride in EtOAc using 4-(dimethylamino)pyridine (DMAP) as a catalyst.^41,42^ The products 4-hydroxybenzonitrile methacrylate **BM**, vanillonitrile methacrylate **VM**, and syringonitrile methacrylate **SM** were readily isolated by straightforward filtration and extraction. **SM** required further purification by aluminum oxide column chromatography to remove residual acid and phenol. The structures and purities of **BM**, **VM**, and **SM** (***3a-c***, respectively) were confirmed by ^1^H NMR spectroscopy. All the signals from **2a** and **2c** were assigned according to literature.^35,43^ The signals from the **BM**, **VM**, and **SM** monomers were also assigned, and no signals from residual phenolic −OH were detected. Today, methacrylic anhydride is produced from fossil sources, but sustainable pathways to methacrylic acid, and subsequently methacrylic anhydride, have been investigated. For example, biobased methacrylic acid has been prepared from both itaconic acid and citric acid^44−47^

The **BM**, **VM**, and **SM** monomers were used to prepare the corresponding homopolymers by free radical polymerization, which is the dominating polymerization procedure employed by industry. The polymerizations were carried out in DMSO solutions during 24 h by thermally initiated free radical polymerization using 2,2′-azobis(2-methylpropionitrile) (AIBN) as the initiator (Scheme 1). In general, toxicological assays of DMSO show low toxicity, and it is considered safe for pharmaceutical usage.^48^ Still, it was recently reported that DMSO induces changes in cellular processes^49^ and is therefore recommended to be substituted.^50,51^ To this extent, green solvents with solubility parameters close to that of DMSO (e.g., acetone, MEK, Cyrene^52^) can be investigated as substitutes for DMSO. The corresponding homopolymers **PBM**, **PVM**, and **PSM** were isolated as white powders after precipitation in methanol, with 49%, 72%, and 93% yields, respectively (Table 1). The variation in the yield may result from differences in the solubility of these highly polar polymers during the polymerization, as the methoxy groups induced a higher solubility in DMSO. The polymerizations were confirmed by ^1^H NMR spectroscopy by observing the disappearance of the alkene signals from the methacrylate moiety. Analysis by size-exclusion chromatography (SEC) showed that **PBM**, **PVM**, and **PSM** reached reasonably high number-average molecular weights (*M*_n_), 36, 23, and 44 kg/mol, corresponding to a degree of polymerization (*X*_n_) of 192, 106 and 178, respectively. The latter two samples were analyzed in both DMF and THF, while the former sample was insoluble in THF and was analyzed only in DMF (Table 1). For both eluents, the calibration was performed using PEO standards. The chromatograms showed a bimodal distribution (Figure S10a) but also some tailing that could be due to interaction with the columns. This may explain the high *Đ* values, 2.1, 2.8, and 2.3 for **PBM**, **PVM**, and **PSM**, respectively. Moreover, the solutions became slightly hazy during the polymerization, which indicated polymer aggregations and a partly heterogeneous polymerization, thus possibly limiting *M*_n_ and increasing *Đ*. Also, we cannot exclude the possibility that the SEC analysis was influenced by interpolymer aggregation facilitated by interactions between the strongly polar nitrile groups. The most important use of the highly polar nitrile-containing monomers is not for the preparation of homopolymers but rather to incorporate smaller amounts into styrenic and acrylic materials to tune and enhance properties such as solvent resistance and shape retention.

**Table 1:** Polymerization and Thermal Data of the Different Benzonitrile-Containing Polymers

Sample name	Nitrile monomer feed (mol %)	Nitrile monomer content in polymer (mol %)^a^	Isolated yield (%)^b^	*M*_n_ (kg/mol)	*Đ*	*T*_d(95%)_ (°C)^e^	*T*_g_ (°C)
PBM	100	100	49	36^d^	2.1^d^	302	150
PVM	100	100	72	23^c^/21^d^	2.8^c^/2.7^d^	303	164
PSM	100	100	93	44^c^/43^d^	2.3^c^/2.1^d^	319	238
							
PSSM-16	10	16	80	19^c^	1.8^c^	317	123
PSSM-25	20	25	70	24^c^	1.7^c^	339	139
PSSM-35	30	35	91	21^c^	1.6^c^	324	152
PSSM-42	40	42	98	24^c^	1.9^c^	322	163
PSSM-50	50	50	89	30^c^	1.8^c^	330	173
							
PSVM-14	10	14	67	20^c^	1.6^c^	340	109
PSVM-28	20	28	72	26^c^	1.7^c^	341	123
PSVM-45	50	45	97	39^c^	1.8^c^	334	128
PMVM-18	10	18	93	85^c^	1.9^c^	261	128
PMVM-41	30	41	63	163^c^	1.5^c^	247	139

a Determined by ^1^H NMR spectroscopy.

b Determined gravimetrically.

c Determined by SEC in THF.

d Determined by SEC in DMF.

e Determined by TGA at 5% weight loss under N_2_.

Here, we studied the effect of the incorporation of the **VM** and **SM** monomers on the properties of polystyrene (PS) and poly(methyl methacrylate) (PMMA). Three series of copolymers were produced using similar conditions as for the homopolymerizations, and we chose to investigate the **VM** more extensively in these copolymerizations because of its higher accessibility. Thus, **VM** was copolymerized with S and MMA to form the **PSVM** and **PMVM** series, respectively (to simplify the acronyms, MMA was shortened to M). In addition, **SM** and S were copolymerized to produce the **PSSM** series (Scheme 1). In contrast to the homopolymerizations, the solutions remained optically clear throughout the copolymerizations. The copolymer contents were determined by ^1^H NMR spectroscopy, and *M*_n_ and *Đ* of the copolymers were determined by SEC (Table 1). A general trend showed that the molar fractions of the **VM** and **SM** monomers were higher in the copolymers than in the feeds, indicating a higher reactivity of these monomers compared to S and MMA in the copolymerizations. The copolymers in the **PSSM** series showed *M*_n_ between 19 and 30 kg/mol, and the **PSVM** series copolymers had *M*_n_ in the range 20 to 39 kg/mol, all with a markedly lower dispersity than the homopolymers (1.6 < *Đ* < 1.9). The SEC curves of these copolymers showed a monomodal distribution (Figure S10b and c) which may result from the much higher solubility in DMSO compared with the homopolymers, due to their much lower nitrile content that reduces the intermolecular interactions. The *M*_n_ values were moderately high and seemed to increase with the **VM** or **SM** content, possibly reflecting the higher reactivity of these monomers compared to S. To increase *M*_n_, the **PMVM** copolymers were produced at a higher monomer concentration. Consequently, the **PMVM** copolymers showed much higher molecular weights with *M*_n_ = 85 and 163 kg/mol, respectively; the SEC curve of **PMVM-41** displayed a bimodal distribution despite showing a lower *Đ* value (Figure S10d).

**Figure 1:**
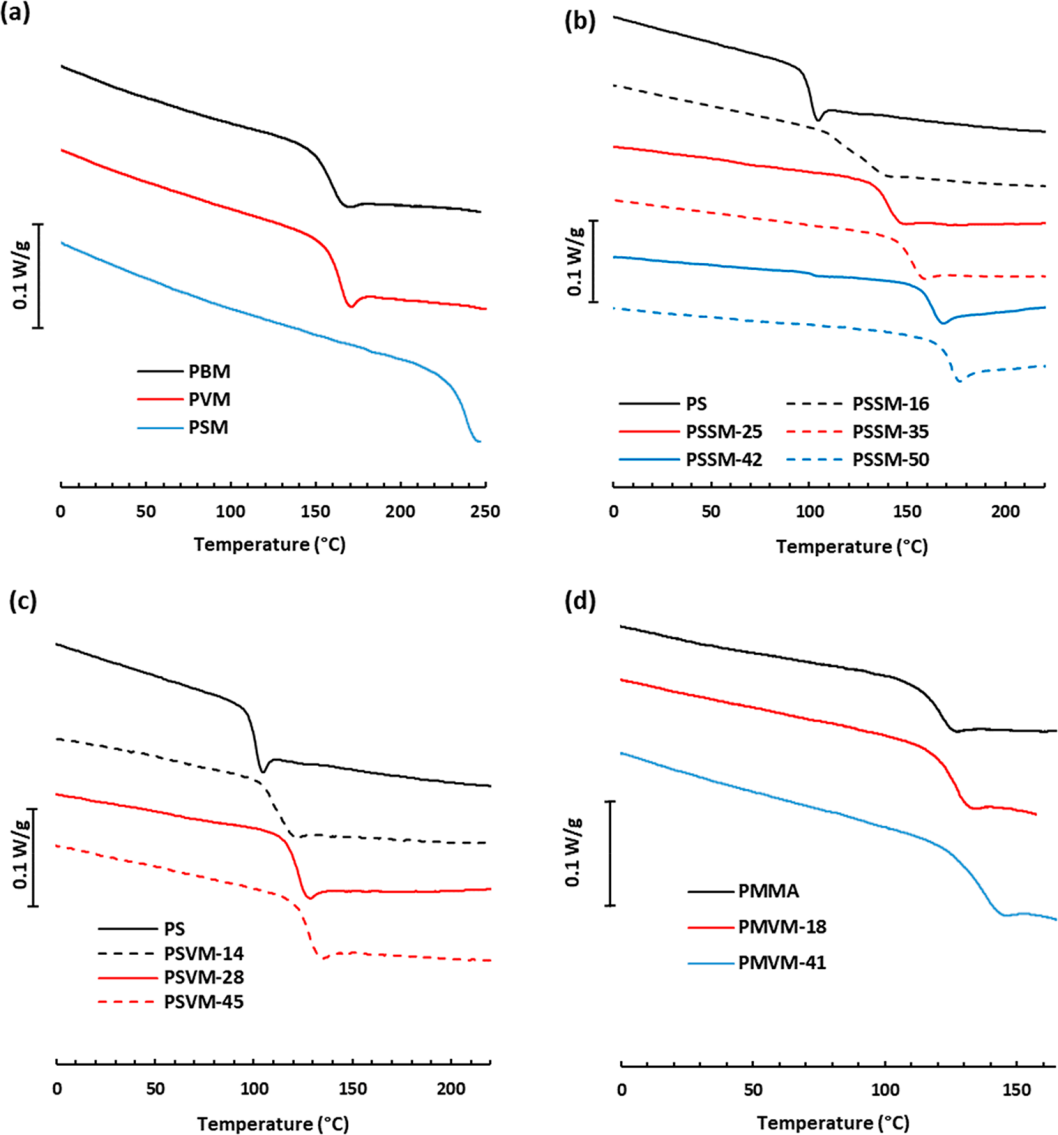
Second heating DSC traces of (a) homopolymers PBM, PVM, and PSM, (b) the PSSM series, (c) the PSVM series, and (d) the PMVM series under N_2_ atmosphere at 10 °C min^–1^.

The thermal transitions of the polymers were characterized by differential scanning calorimetry (DSC) measurements. As expected, all the polymers were fully amorphous and displayed single glass transitions (Figure 1a). The *T*_g_ values measured for the homopolymers were 150, 164, and 238 °C for **PBM**, **PVM**, and **PSM**, respectively (Table 1). Consequently, *T*_g_ increased markedly with the number of methoxy groups present on the aromatic ring, which corroborates previous observations on methoxy-substituted polymethacrylates.^9,53,54^ The methoxy groups most probably prevent the rotation of the aromatic ring, thus reducing the segmental mobility of the polymer chains. Notably, the opposite effect has been reported for vanillin-based epoxy networks, where the presence of a methoxy group on the aromatic ring resulted in a decreased *T*_g_.^55^ The present polymers show improved thermal properties compared to neat PS (*T*_g_ = 100 °C) and PMMA (*T*_g_ = 105 °C). Moreover, both **PVM** and **PSM** show significantly higher *T*_g_ values than corresponding non-nitrilated polymers. Emerson et al. reported *T*_g_ = 129 and 201 °C for vanillin- and syringaldehyde-based polymethacrylates, respectively.^53^ The higher *T*_g_ values of the present polymers can be explained by the very high polarity of the nitrile group in comparison to the aldehyde group (μ = 4.18 and 2.77 D for benzonitrile and benzaldehyde, respectively).^56,57^ Hence, the nitrile-containing polymers reached significantly higher *T*_g_ values compared to other methoxy-substituted polymethacrylates (Table S2).^25−27,53^ The very high *T*_g_ values displayed by **PVM** and **PSM** motivated the study on the effect of the incorporating **VM** and **SM** into S and MMA copolymers. As expected, the DSC traces of the copolymers in the **PSSM** series presented gradually increasing values with the **SM** content, with *T*_g_ = 100, 123, and 173 °C for PS, **PSSM-16**, and **PSSM-50**, respectively (Figure 1b and Table 1). Similarly, the **PSVM** copolymers showed *T*_g_ values which gradually increased from 109 °C for **PSVM-14** to 128 °C for **PSVM-45** (Figure 1c and Table 1). Finally, the **PMVM** copolymers displayed *T*_g_ values which gradually increased from 121 to 128 and 139 °C for PMMA, **PMVM-18**, and **PMVM-41**, respectively (Figure 1d and Table 1). Hence, the *T*_g_ was observed to increase with the nitrile methacrylate content in all the copolymer series. Notably, the PMMA synthesized using the same protocol as the **PMVM** series showed a *T*_g_ of 121 °C, which was higher than the usually reported 105 °C for neat PMMA. As a matter of fact, PMMA obtained by free radical polymerization usually possess slightly syndiotactic-rich configuration (*rr* ∼ 60–70%), thus increasing *T*_g_.^58^ A ^1^H NMR analysis of the present sample revealed a content of ca. 60% of *rr* triads, indicating a degree of syndiotacticity that can explain the higher *T*_g_ observed (Figure S6).

**Figure 2:**
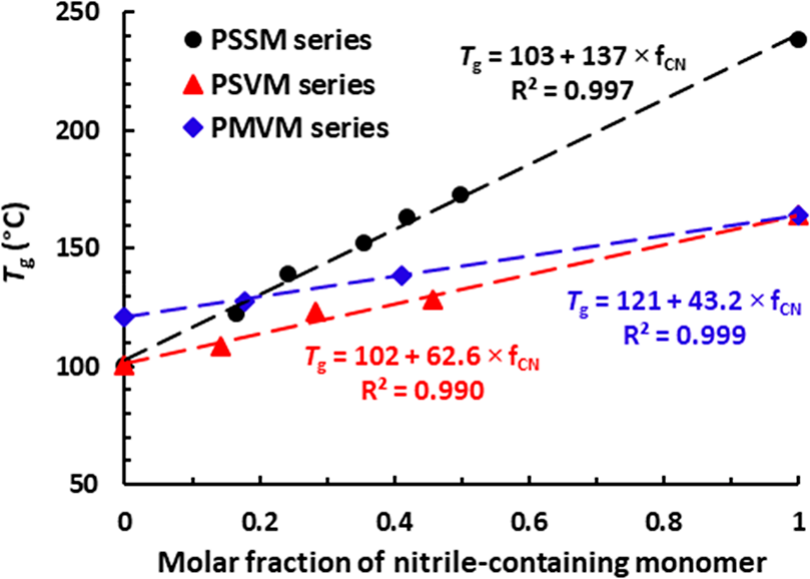
*T*_g_ of the homopolymers and copolymers versus the molar fraction of nitrile-containing monomer. The dashed lines indicate fittings to eq 1.

As shown in Figure 2, the variation of *T*_g_ with the **VM** and **SM** content, respectively, followed a linear trend for the **PSSM**, **PSVM**, and **PMVM** series in the form
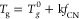
1where *f*_CN_ is the molar fraction of the nitrile monomer. This indicates that the copolymers follow the rule of mixtures. In Figure S9, the variation of 1/*T*_g_ with the weight fraction of **VM** and **SM**, respectively, followed a linear trend for the **PSSM**, **PSVM**, and **PMVM** copolymer series, indicating that they also seemed to follow the Flory–Fox equation. In conclusion, the results showed that the *T*_g_ of styrenic and methacrylic materials can be tuned and enhanced in a controllable way by incorporating predetermined amounts of the biobased nitrile monomers. This is of particular interest for applications where, for example, high-temperature shape retention is required.

The thermal stability of the polymers was analyzed by thermogravimetric analysis (TGA) to study their thermal decomposition under N_2_ atmosphere. The TGA traces showed that the **PBM**, **PVM**, and **PSM** homopolymers decomposed in one step at *T*_d,95%_ = 302, 303, and 319 °C, respectively (Figures S7a and S8a, Table 1). *T*_d,95%_ seemed to increase with the number of methoxy groups on the aromatic ring, which might be explained by the increasing *T*_g_ (i.e., higher melt viscosity) of the polymers. Both **PVM** and **PSM** showed considerably higher *T*_d,95%_ values than the corresponding non-nitrilated vanillin- and syringaldehyde-based polymethacrylates reported by Holmberg and co-workers with *T*_d,95%_ = 264 and 303 °C, respectively.^26,59^ This demonstrated that the introduction of the nitrile group significantly enhanced the thermal stability. Furthermore, the *T*_d,95%_ value reached to approximately 150 and ∼140 °C above *T*_g_ for **PBM** and **PVM**, respectively, and to about 80 °C above the *T*_g_ of **PSM**. This implied that the thermal window of **PBM** and **PVM** is sufficiently high to enable melt processing, which is generally performed ca. 30–70 °C above the *T*_g_ of amorphous polymers. The narrower window of **PSM** indicated that the melt processing of this polymer is more restricted. The TGA traces of the copolymers are reported in Figure S7b–d. Here, *T*_d,95%_ varied in the quite narrow range between 317 and 330 °C for the **PSSM** copolymers, 334 and 341 °C for the **PSVM** copolymers, and 247 and 261 °C for the **PMVM** copolymers (Table 1). No obvious trend between copolymer compositions and *T*_d,95%_ was identified. For all the copolymers, the difference between *T*_d,95%_ and *T*_g_ values was higher than 100 °C, which indicated that the copolymers may be melt processable.

**Figure 3:**
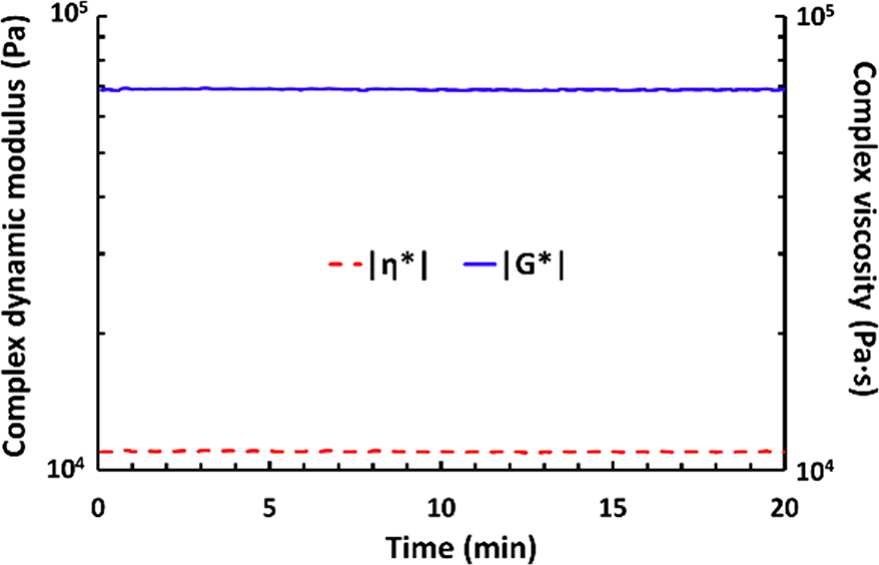
Dynamic melt rheology data of the complex dynamic shear modulus |G*| and complex viscosity |η*| for copolymer **PSVM-14** at 150 °C during 20 min at 0.1% strain.

Dynamic melt rheology was carried out in order to further investigate the processability of the copolymers. Thus, sample **PSVM-14** was kept at 150 °C during 20 min under a sinusoidal stress. As can be seen in Figure 3, |G*| and |η*| remained constant throughout the experiment, which hinted that the sample did not suffer from any significant chain scission or cross-linking reactions and, hence, that **PSVM-14** was stable under these conditions. This showed that the **VM** moiety did not degrade when exposed to a typical melt processing time and temperature, implying that the **VM** copolymers are melt processable.

The solvent resistance of the different polymers was probed by investigating the solubility in a wide range of solvents (Table S1) chosen according to their solubility parameter (δ), as well as their hydrogen bonding capacity. The highly polar nitrile-containing homopolymers were insoluble in strongly hydrogen-bonding solvents such as H_2_O, MeOH, and 1-BuOH. They were soluble in DMSO but insoluble in Et_2_O and toluene. As expected, PS was fully soluble in these latter two solvents, but the copolymers in the **PSVM** and **PSSM** series became nonsoluble in Et_2_O and toluene as the nitrile-containing methacrylate content increased. Thus, **PSVM-14** and **PSSM-16** were insoluble in Et_2_O, while **PSVM-45** and **PSSM-25** were insoluble in toluene. The introduction of **VM** and **SM** thus gave styrenic materials with solvent resistance characteristics comparable to SAN.^60^ While PMMA was soluble in acetonitrile (ACN), **PVM** was not. Thus, the copolymers in the **PMVM** series became nonsoluble in ACN (as observed for **PMVM-41**). Consequently, both the **VM** and the **SM** monomers allowed efficient tuning of the solvent resistance of styrenic and acrylic polymer materials.

## Conclusion

In summary, nitrile-containing methacrylates were readily prepared by nitrilation of corresponding lignin-inspired aldehyde-functional building blocks such as vanillin and syringaldehyde using environmentally benign methods, followed by methacrylation. The presence of the highly polar nitrile group and methoxy groups on the phenolic ring contribute to the exceptionally high *T*_g_ reached by the corresponding polymethacrylates. Copolymerizations to incorporate the monomers into styrenics and acrylics gave materials with enhanced and controllable *T*_g_ values and solvent resistance. In addition, thermal and rheological characterizations indicated that polymers containing vanillonitrile methacrylate**VM** were melt processable. The use of the lignin-inspired nitrile-containing methacrylates represents a new strategy to prepare high-performance biobased polymer materials with high dimensional stability and solvent resistance. Especially, vanillonitrile methacrylate holds great promise for applications within, for example, packaging, coating, and high-performance plastics applications because of the accessibility and potentially low cost of vanillin.

## Supporting Information Available

Experimental section containing description of materials and instruments (^1^H NMR, SEC, TGA, DSC, rheology) and detailed experimental descriptions of all reported precursors, monomers, polymers and copolymers; ^1^H NMR spectra of **2c**, **BM**, **VM**, **SM**, **PSSM-16**, and **PMMA**; TGA data of the homopolymers and copolymers; Fox–Flory plot of the copolymers; SEC chromatograms of the homopolymers and copolymers; solubility data of the homopolymers and copolymers; table with structures and *T*_g_ of reported lignin-based polymethacrylates (PDF)
