# Supplementary material for: Lignin-Inspired Polymers with High Glass Transition Temperature and Solvent Resistance from 4-Hydroxybenzonitrile, Vanillonitrile, and Syringonitrile Methacrylates
Source: ACS Sustain Chem Eng. 2021 Dec 7;9(50):16874–80. doi: 10.1021/acssuschemeng.1c07048 (PMC8693774; doi:10.1021/acssuschemeng.1c07048)
Supplement: Supplementary file 1 — sc1c07048_si_001.pdf [file sc1c07048_si_001.pdf]

Supporting Information

Lignin-Inspired Polymers with High Glass Transition Temperature and Solvent Resistance from 4-hydroxybenzo-, Vanillo- and Syringonitrile Methacrylates

Olivier Bonjour[1], Hannes Nederstedt[1], Monica V. Arcos-Hernandez[1], Siim Laanesoo[2], Lauri Vares[2], Patric Jannasch[1,2,]*

[1] Centre for Analysis and Synthesis, Department of Chemistry, Lund University, P.O. Box 124, SE-22100 Lund, Sweden

[2] Institute of Technology, University of Tartu, Nooruse 1, Tartu 50411, Estonia

*Corresponding author e-mail: patric.jannasch@chem.lu.se

Number of pages in Supporting Information: 22

Number of tables in Supporting Information: 2

Number of figures in Supporting information: 10

Table of Contents

Experimental section 3
Materials 3
Analytical Methods 3
Thermogravimetry and calorimetry 3
Melt rheology 3
Synthesis of compounds 3
4-Hydroxybenzonitrile 2a 3
4-Hydroxy-3,5-dimethoxybenzonitrile (syringonitrile) 2c 4
4-Cyanophenyl methacrylate (benzonitrile methacrylate) BM 4
4-Cyano-2-methoxyphenyl methacrylate (vanillonitrile methacrylate) VM 4
4-Cyano-2,6-dimethoxyphenyl methacrylate (syringonitrile methacrylate) SM 5
Poly(4-cyanophenyl methacrylate) [poly(benzonitrile methacrylate)] PBM 5
Poly(4-cyano-2-methoxyphenyl methacrylate) [poly(vanillonitrile methacrylate)] PVM 5
Poly[styrene-co-(4-cyano-2-methoxyphenyl methacrylate)] PSVM-14 5
Poly[styrene-co-(4-cyano-2-methoxyphenyl methacrylate)] PSVM-28 6
Poly[styrene-co-(4-cyano-2-methoxyphenyl methacrylate)] PSVM-45 6
Poly(4-cyano-2,6-dimethoxyphenyl methacrylate) [poly(syringonitrile methacrylate)] PSM 6
Poly[styrene-co-(4-cyano-2,6-dimethoxyphenyl methacrylate)] PSSM-16 6
Poly[styrene-co-(4-cyano-2,6-dimethoxyphenyl methacrylate)] PSSM-25 7
Poly[styrene-co-(4-cyano-2,6-dimethoxyphenyl methacrylate)] PSSM-35 7
Poly[styrene-co-(4-cyano-2,6-dimethoxyphenyl methacrylate)] PSSM-42 7
Poly[styrene-co-(4-cyano-2,6-dimethoxyphenyl methacrylate)] PSSM-50 7
Poly[(methyl 2-methylpropenoate)-co-(4-cyano-2-methoxyphenyl methacrylate)] PMVM-18 8
Poly[(methyl 2-methylpropenoate)-co-(4-cyano-2-methoxyphenyl methacrylate)] PMVM-41 8
NMR Spectra 9
TGA Traces 15
Flory-Fox plot 17
SEC Curves 18
Evaluation of the Solubility of Polybenzonitrile methacrylates 20
Summary of previously reported T_gs of lignin-based polymethacrylates. 21

Experimental section

Materials

All reagents and solvents were obtained from commercial sources and used without further purification. The monomer synthesis steps were monitored by thin-layer chromatography (TLC, silica gel 60 F_{254}), and TLC plates were visualized using a UV lamp. Aluminum oxide (active basic, particle size 0.063-0.200 mm (70-230 mesh ASTM)) was used in the flash chromatography.

Analytical Methods

Nuclear Magnetic Resonance (NMR) spectroscopy was performed on a Bruker DR X400 spectrometer at 400.13 MHz proton frequency and 100.62 MHz carbon frequency. The formation of the polymers was determined by the decrease of the double bond signals of the methacrylate moiety. The molecular weight of the polymers was determined by Size Exclusion Chromatography (SEC) using THF or DMF/0.05 M LiBr as eluent at 40 °C. The instrument included Shodex columns coupled in series (KF-805, -804 and -802.5 for THF and KD-804 and -802.5 for DMF) placed in a Shimadzu CTO-20A prominence column oven, a Shimadzu RID-20A refractive index detector, with Shimadzu LabSolution software. Calibration was done by using poly(ethylene oxide) standards (M_n = 3.86, 21.16, 49.39 and 96.1 kg/mol). All samples were run at an elution rate of 1 mL/min.

Thermogravimetry and calorimetry

Thermogravimetric analysis (TGA) was performed on a TA Instruments TGA Q500. Samples from 1-6 mg were heated to 600 °C at a rate of 10 °C/min under nitrogen flux (60 mL/min). The thermal decomposition temperature ($T_{d,95\%}$) was determined at 5% loss of the total weight. Differential scanning calorimetry (DSC) measurements were performed on TA Instruments DSC Q2000. Samples from 1-10 mg were first heated up to 170-250 °C (depending on their respective thermal decomposition temperature) at a rate of 10 °C/min. They were kept at this temperature for 2 min, then cooled down to -50 °C and kept isothermal for 2 min, before being heated up again to the previous temperature, at the same heating rate.

Melt rheology

Dynamic rheology measurements were performed with TA Instruments Advanced Rheometer AR2000 ETC. The experiments were made using parallel plates (Ø = 15 mm). Discs of PSVM-14, PSVM-28, PSSM-25 and PMVM-18 (Ø = 15 mm, t = 1 mm) were hot-pressed between two aluminum plates using a hydraulic press (Specac, GS15011) at 150 °C during 5 min, and finally cooled to room temperature. A time sweep was carried out during 8 h at 150 °C, at a frequency of 1 Hz at 0.1% strain, which was within the linear viscoelastic region.

Synthesis of compounds

4-Hydroxybenzonitrile 2a

To a 250 mL round-bottom flask equipped with a magnetic stirrer, 4-hydroxybenzaldehyde **1a** (4.000 g, 32.8 mmol, 1.0 equiv.) and hydroxylamine-*O*-sulfonic acid (4.075 g, 36.0 mmol, 1.1 equiv.) were dissolved in a mixture of water (160 mL) and acetic acid (1.970 g, 32.8 mmol, 1.0 equiv.) cooled with an ice bath. The reaction mixture was stirred at 50 °C for 6 h. After completion, the reaction mixture was quenched by

a 10% $NaHCO_3$ aqueous solution, until no gas release was observed. The product was extracted by EtOAc (3 × 60 mL). The organic phases were gathered, dried over anhydrous sodium sulfate, filtered and concentrated with a rotary evaporator. The residue was purified by recrystallization in heptane. The product was dried carefully under vacuum to afford 3.180 g of a light brown powder (yield: 82%). ^{1}H NMR (400.13 MHz, $CDCl_3$) δ, ppm: 6.30 (s, 1H), 6.91-6.95 (m, 2H), 7.54-7.58 (m, 2H), melting temperature: T_m = 112 °C.

4-Hydroxy-3,5-dimethoxybenzonitrile (syringonitrile) 2c

To a 250 mL round-bottom flask equipped with a magnetic stirrer, 4-hydroxy-3,5-dimethoxybenzaldehyde ***1c*** (5.000 g, 27.4 mmol, 1.0 equiv.) and hydroxylamine-*O*-sulfonic acid (3.414 g, 30.2 mmol, 1.1 equiv.) were dissolved in a mixture of water (50 mL) and acetic acid (1.648 g, 27.4 mmol, 1.0 equiv.) cooled with an ice bath. The reaction mixture was stirred at 50 °C for 6 h. After completion, the reaction mixture was quenched by 10% $NaHCO_3$ until no gas release was observed. The product was extracted by EtOAc (3 × 30 mL). The organic phases were gathered, dried over anhydrous sodium sulfate, filtered and concentrated with a rotary evaporator. The residue was purified by recrystallization in heptane. The product was dried carefully under vacuum to afford 3.874 g of a light brown powder (yield: 79%). ^{1}H NMR (400.13 MHz, $CDCl_3$) δ, ppm: 3.90 (s, 6H), 6.02 (s, 1H), 6.85 (s, 2H); melting temperature: T_m = 128 °C.

4-Cyanophenyl methacrylate (benzonitrile methacrylate)* BM *3a

To a 25 mL round-bottom flask equipped with a magnetic stirrer, 4-hydroxybenzonitrile ***2a*** (2.90 g, 24.3 mmol, 1.0 equiv.) and DMAP (0.06 g, 0.49 mmol, 0.02 equiv.) were dissolved in EtOAc (4.0 mL). The flask was sealed and purged with vacuum/N_2 cycles. Methacrylic anhydride (3.79 g, 24.6 mmol, 1.01 equiv.) was added dropwise to the reaction mixture. The flask was purged again as described above, and the reaction mixture was then heated at 65 °C for 2 h. The obtained precipitate was filtered and washed with EtOAc (25 mL). The filtrate was washed with a saturated $NaHCO_3$ aqueous solution, until no gas release was observed. The organic phase was then washed with sodium hydroxide (2.5 M, 25 mL), hydrochloric acid (1 M, 25 mL) and water (25 mL). The organic phase was dried over anhydrous sodium sulfate, filtered and concentrated with a rotary evaporator. The product was dried carefully under vacuum to afford 3.180 g (from precipitate and filtrate) of a white powder (yield: 70%). ^{1}H NMR (400.13 MHz, DMSO-d_6) δ, ppm: 2.00 (m, 3H), 5.93-5.95 (m, 1H), 6.30-6.31 (m, 1H), 7.41-7.45 (m, 2H), 7.91-7.95 (m, 2H), ^{13}C NMR (100.62 MHz, DMSO-d_6) δ, ppm: 17.9, 108.8, 118.4, 123.3, 128.5, 134.0, 134.9, 154.1, 164.7; 1H NMR (400 MHz, CDCl3) δ, ppm: 2.06 (m, 3H), 5.82 (m, 1H), 6.38 (m, 1H), 7.25-7.29 (m, 2H), 7.69-7.72 (m, 2H), ^{13}C NMR (100.62 MHz, $CDCl_3$) δ, ppm: 18.4, 109.8, 118.4, 122.9, 128.5, 133.8, 135.4, 154.4, 165.0; melting temperature: decomposition before reaching melting

4-Cyano-2-methoxyphenyl methacrylate (vanillonitrile methacrylate)* VM *3b

To a 100 mL round-bottom flask equipped with a magnetic stirrer, vanillonitrile ***2b*** (21.08 g, 0.14 mol, 1.0 equiv.) and DMAP (0.35 g, 2.8 mmol, 0.02 equiv.) were dissolved in EtOAc (40 mL). The flask was sealed and purged with vacuum/N_2 cycles. Methacrylic anhydride (22.01 g, 0.14 mol, 1.01 equiv.) was added dropwise to the reaction mixture. The flask was purged again as described above, and the reaction mixture was then heated at 55 °C for 24 h. After completion, EtOAc (25 mL) were added to the reaction mixture, which was subsequently washed with a saturated $NaHCO_3$ aqueous solution, until no gas release was observed. The organic phase was then washed with sodium hydroxide (2.5 M, 25 mL), hydrochloric acid (1 M, 25 mL) and water (25 mL). The organic phase was dried over anhydrous sodium sulfate, filtered and

concentrated with a rotary evaporator. The product was dried carefully under vacuum to afford 29.197 g of a white powder (yield: 89%). ^{1}H NMR (400.13 MHz, DMSO-d$_6$) δ, ppm: 1.99 (m, 3H), 3.84 (s, 3H), 5.93-5.94 (m, 1H), 6.29 (m, 1H), 7.37-7.39 (d, J = 8.1 Hz, 1H), 7.48-7.50 (dd, J = 1.8 and 8.2 Hz, 1H), 7.67 (d, J = 1.8 Hz, 1H), ^{13}C NMR (100.62 MHz, DMSO-d$_6$) δ, ppm: 18.0, 56.6, 109.7, 116.5, 118.4, 124.3, 125.5, 128.6, 134.5, 143.4, 151.5, 164.2; ^{1}H NMR (400 MHz, $CDCl_3$) δ, ppm: 2.06 (m, 3H), 3.86 (s, 3H), 5.79-5.81 (m, 1H), 6.37 (m, 1H), 7.16-7.18 (d, J = 8.16 Hz, 1H), 7.21 (d, J = 1.77 Hz, 1H), 7.29-7.31 (dd, J = 1.80 and 8.12 Hz, 1H), ^{13}C NMR (100.62 MHz, $CDCl_3$) δ, ppm: 18.5, 56.4, 110.6, 115.8, 118.5, 124.2, 125.5, 128.3, 135.2, 144.1, 152.0, 164.7; melting temperature: decomposition before reaching melting

4-Cyano-2,6-dimethoxyphenyl methacrylate (syringonitrile methacrylate)* SM *3c

To a 100 mL round-bottom flask equipped with a magnetic stirrer, 4-hydroxy-3,5-dimethoxybenzonitrile ***2c*** (2.862 g, 15.97 mmol, 1.0 equiv.) and DMAP (39.4 mg, 0.323 mmol, 0.02 equiv.) were dissolved in EtOAc (26 mL). The flask was sealed and purged with vacuum/N_2 cycles. Methacrylic anhydride (2.487 g, 16.13 mmol, 1.01 equiv.) was added dropwise to the reaction mixture. The flask was purged again as described above, and the reaction mixture was then heated at 65 °C for 24 h. The precipitate was filtered and washed with cold EtOAc (25 mL). SM was further purified by aluminum oxide column chromatography, with heptane as eluent, to remove residual acid and phenol. The product was dried carefully under vacuum to afford 2.915 g of a white powder (yield: 74%). ^{1}H NMR (400.13 MHz, $CHCl_3$) δ, ppm: 2.06 (m, 3H), 3.83 (s, 6H), 5.78 (m, 1H), 6.37 (m, 1H), 6.89 (s, 2H), ^{13}C NMR (100.62 MHz, $CDCl_3$) δ, ppm: 18.5, 56.6, 109.1, 109.9, 118.7, 128.1, 133.1, 135.0, 153.0, 164.5; melting temperature: decomposition before reaching melting

***Poly(4-cyanophenyl methacrylate) [poly(benzonitrile methacrylate)]* PBM**

In a schlenk flask, 10 mL of acetonitrile were degassed with vacuum/N_2 cycles at 40 °C. To a 25 mL schlenk flask equipped with a magnetic stirrer, 4-cyanophenyl methacrylate **BM *3a*** (0.650 g, 3.475 mmol, 1.0 equiv.) were dissolved in degassed acetone (4.00 mL) and degassed again as before. In a vial, azobisisobutyronitrile (AIBN) (3.0 mg) were dissolved in degassed acetonitrile (2 mL). 1 mL of the AIBN solution is introduced in the flask with the dissolved monomer, and degassed again as before. The reaction mixture was then heated at 60 °C for 24 h. The resulting material was precipitated in MeOH, and the white powder was filtered off and washed repeatedly with MeOH before drying at 50 °C under vacuum, yielding 0.316 g (49%).

***Poly(4-cyano-2-methoxyphenyl methacrylate) [poly(vanillonitrile methacrylate)]* PVM**

In a schlenk flask, 6 mL of DMSO were degassed with vacuum/N_2 cycles at 40 °C. To a 25 mL schlenk flask equipped with a magnetic stirrer, 4-cyano-2-methoxyphenyl methacrylate **VM *3b*** (0.500 g, 2.302 mmol, 1.0 equiv.) were dissolved in degassed DMSO (2.00 mL) and degassed again as before. In a vial, azobisisobutyronitrile (AIBN) (3.22 mg) were dissolved in degassed DMSO (2 mL). 1.00 mL of the AIBN solution were introduced in the flask with the dissolved monomer, and degassed again as before. The reaction mixture was then heated at 60 °C for 24 h. The resulting material was precipitated in MeOH, and the white powder was filtered off and washed repeatedly with MeOH before drying at 50 °C under vacuum, yielding 0.360 g (72%).

***Poly[styrene-co-(4-cyano-2-methoxyphenyl methacrylate)]* PSVM-14**

In a schlenk flask, 10 mL of DMSO were degassed with vacuum/N_2 cycles at 40 °C. To a 25 mL schlenk flask equipped with a magnetic stirrer, 4-cyano-2-methoxyphenyl methacrylate **VM *3b*** (0.094 g, 0.433

mmol) and styrene (0.406 g, 3.897 mmol) were dissolved in degassed DMSO (4.56 mL) and degassed again as before. In a vial, azobisisobutyronitrile (AIBN) (20 mg) were dissolved in degassed DMSO (2 mL). 1.42 mL of the AIBN solution were introduced in the flask with the dissolved monomers, and degassed again as before. The reaction mixture was then heated at 60 °C for 24 h. The resulting material was precipitated in MeOH. The formed white powder was filtered off and washed repeatedly with MeOH before drying at 50 °C under vacuum, yielding 0.337 g (67%).

***Poly[styrene-co-(4-cyano-2-methoxyphenyl methacrylate)]* PSVM-28**

In a schlenk flask, 10 mL of DMSO were degassed with vacuum/N_2 cycles at 40 °C. To a 25 mL schlenk flask equipped with a magnetic stirrer, 4-cyano-2-methoxyphenyl methacrylate **VM *3b*** (0.171 g, 0.789 mmol) and styrene (0.329 g, 3.155 mmol) were dissolved in degassed DMSO (4.20 mL) and degassed again as before. In a vial, azobisisobutyronitrile (AIBN) (20 mg) were dissolved in degassed DMSO (2 mL). 1.29 mL of the AIBN solution were introduced in the flask with the dissolved monomers, and degassed again as before. The reaction mixture was then heated at 60 °C for 24 h. The resulting material was precipitated in MeOH, and the white powder was filtered off and washed repeatedly with MeOH before drying at 50 °C under vacuum, yielding 0.361 g (72%).

***Poly[styrene-co-(4-cyano-2-methoxyphenyl methacrylate)]* PSVM-45**

In a schlenk flask, 10 mL of DMSO were degassed with vacuum/N_2 cycles at 40 °C. To a 25 mL schlenk flask equipped with a magnetic stirrer, 4-cyano-2-methoxyphenyl methacrylate **VM *3b*** (0.338 g, 1.556 mmol) and styrene (0.162 g, 1.556 mmol) were dissolved in degassed DMSO (3.42 mL) and degassed again as before. In a vial, azobisisobutyronitrile (AIBN) (20 mg) were dissolved in degassed DMSO (2 mL). 1.02 mL of the AIBN solution were introduced in the flask with the dissolved monomers, and degassed again as before. The reaction mixture was then heated at 60 °C for 24 h. The resulting material was precipitated in MeOH, and the white powder was filtered off and washed repeatedly with MeOH before drying at 50 °C under vacuum, yielding 0.487 g (97%).

***Poly(4-cyano-2,6-dimethoxyphenyl methacrylate) [poly(syringonitrile methacrylate)]* PSM**

In a schlenk flask, 6 mL of DMSO were degassed with vacuum/N_2 cycles at 40 °C. To a 25 mL schlenk flask equipped with a magnetic stirrer, 4-cyano-2,6-dimethoxyphenyl methacrylate **SM *3c*** (0.500 g, 2.022 mmol, 1.0 equiv.) were dissolved in degassed DMSO (2.56 mL) and degassed again as before. In a vial, azobisisobutyronitrile (AIBN) (30 mg) were dissolved in degassed DMSO (2 mL). 0.44 mL of the AIBN solution were introduced in the flask with the dissolved monomer, and degassed again as before. The reaction mixture was then heated at 60 °C for 24 h. The resulting material was precipitated in MeOH, and the white powder was filtered off and washed repeatedly with MeOH before drying at 50 °C under vacuum, yielding 0.463 g (93%).

***Poly[styrene-co-(4-cyano-2,6-dimethoxyphenyl methacrylate)]* PSSM-16**

In a schlenk flask, 10 mL of DMSO were degassed with vacuum/N_2 cycles at 40 °C. To a 25 mL schlenk flask equipped with a magnetic stirrer, 4-cyano-2,6-dimethoxyphenyl methacrylate **SM *3c*** (0.104 g, 0.422 mmol) and styrene (0.396 g, 3.798 mmol) were dissolved in degassed DMSO (2.5 mL) and degassed again as before. In a vial, azobisisobutyronitrile (AIBN) (83 mg) were dissolved in degassed DMSO (2.5 mL). 0.5 mL of the AIBN solution were introduced in the flask with the dissolved monomers, and degassed again as before. The reaction mixture was then heated at 60 °C for 24 h. The resulting material was precipitated

in MeOH. The formed white powder was filtered off and washed repeatedly with MeOH before drying at 50 °C under vacuum, yielding 0.398 g (80%).

***Poly[styrene-co-(4-cyano-2,6-dimethoxyphenyl methacrylate)]* PSSM-25**

In a schlenk flask, 10 mL of DMSO were degassed with vacuum/N_2 cycles at 40 °C. To a 25 mL schlenk flask equipped with a magnetic stirrer, 4-cyano-2,6-dimethoxyphenyl methacrylate **SM *3c*** (0.186 g, 0.753 mmol) and styrene (0.314 g, 3.013 mmol) were dissolved in degassed DMSO (2.5 mL) and degassed again as before. In a vial, azobisisobutyronitrile (AIBN) (83 mg) were dissolved in degassed DMSO (2.5 mL). 0.5 mL of the AIBN solution were introduced in the flask with the dissolved monomers, and degassed again as before. The reaction mixture was then heated at 60 °C for 24 h. The resulting material was precipitated in MeOH, and the white powder was filtered off and washed repeatedly with MeOH before drying at 50 °C under vacuum, yielding 0.351 g (70%).

***Poly[styrene-co-(4-cyano-2,6-dimethoxyphenyl methacrylate)] PSSM*-35**

In a schlenk flask, 10 mL of DMSO were degassed with vacuum/N_2 cycles at 40 °C. To a 25 mL schlenk flask equipped with a magnetic stirrer, 4-cyano-2,6-dimethoxyphenyl methacrylate **SM *3c*** (0.252 g, 1.019 mmol) and styrene (0.248 g, 2.379 mmol) were dissolved in degassed DMSO (2.5 mL) and degassed again as before. In a vial, azobisisobutyronitrile (AIBN) (83 mg) were dissolved in degassed DMSO (2.5 mL). 0.5 mL of the AIBN solution were introduced in the flask with the dissolved monomers, and degassed again as before. The reaction mixture was then heated at 60 °C for 24 h. The resulting material was precipitated in MeOH, and the white powder was filtered off and washed repeatedly with MeOH before drying at 50 °C under vacuum, yielding 0.453 g (91%).

***Poly[styrene-co-(4-cyano-2,6-dimethoxyphenyl methacrylate)]* PSSM-42**

In a schlenk flask, 10 mL of DMSO were degassed with vacuum/N_2 cycles at 40 °C. To a 25 mL schlenk flask equipped with a magnetic stirrer, 4-cyano-2,6-dimethoxyphenyl methacrylate **SM *3c*** (0.306 g, 1.239 mmol) and styrene (0.194 g, 1.859 mmol) were dissolved in degassed DMSO (2.5 mL) and degassed again as before. In a vial, azobisisobutyronitrile (AIBN) (83 mg) were dissolved in degassed DMSO (2.5 mL). 0.5 mL of the AIBN solution were introduced in the flask with the dissolved monomers, and degassed again as before. The reaction mixture was then heated at 60 °C for 24 h. The resulting material was precipitated in MeOH, and the white powder was filtered off and washed repeatedly with MeOH before drying at 50 °C under vacuum, yielding 0.488 g (98%).

***Poly[styrene-co-(4-cyano-2,6-dimethoxyphenyl methacrylate)]* PSSM-50**

In a schlenk flask, 10 mL of DMSO were degassed with vacuum/N_2 cycles at 40 °C. To a 25 mL schlenk flask equipped with a magnetic stirrer, 4-cyano-2,6-dimethoxyphenyl methacrylate **SM *3c*** (0.352 g, 1.423 mmol) and styrene (0.148 g, 1.423 mmol) were dissolved in degassed DMSO (2.5 mL) and degassed again as before. In a vial, azobisisobutyronitrile (AIBN) (83 mg) were dissolved in degassed DMSO (2.5 mL). 0.5 mL of the AIBN solution were introduced in the flask with the dissolved monomers, and degassed again as before. The reaction mixture was then heated at 60 °C for 24 h. The resulting material was precipitated in MeOH, and the white powder was filtered off and washed repeatedly with MeOH before drying at 50 °C under vacuum, yielding 0.433 g (89%).

***Poly[(methyl 2-methylpropenoate)-co-(4-cyano-2-methoxyphenyl methacrylate)]* PMVM-18**

In a schlenk flask, 10 mL of DMSO were degassed with vacuum/N_2 cycles at 40 °C. In another schlenk flask, 20 mL of methyl methacrylate (MMA) were degassed with the same procedure. To a 25 mL schlenk flask equipped with a magnetic stirrer, 4-cyano-2-methoxyphenyl methacrylate **VM *3b*** (0.191 mg, 0.880 mmol), MMA (0.63 mL) and DMSO (1.00 mL) were placed under N_2 atmosphere. In a volumetric flask (10 mL), azobisisobutyronitrile (AIBN) (0.500 mg) were dissolved in degassed MMA. 0.23 mL of the AIBN solution were introduced in the reaction vessel and degassed again as before (MMA 0.809 g, 7.920 mmol in total; AIBN 11.56 mg, 0.0704 mmol, 0.8 mol%). The reaction mixture was then heated at 60 °C for 17 h. The resulting material was dissolved in $CHCl_3$ (20 mL) and then precipitated in MeOH (200 mL). The formed white fibers were filtered off on Büchner and washed repeatedly with MeOH. The solid was dissolved again with $CHCl_3$ and concentrated with a rotary evaporator. The residue was dried at 50 °C under vacuum, yielding 0.93 g (93%).

***Poly[(methyl 2-methylpropenoate)-co-(4-cyano-2-methoxyphenyl methacrylate)]* PMVM-41**

In a schlenk flask, 10 mL of DMSO were degassed with vacuum/N_2 cycles at 40 °C. In another schlenk flask, 20 mL of methyl methacrylate (MMA) were degassed with the same procedure. To a 25 mL schlenk flask equipped with a magnetic stirrer, 4-cyano-2-methoxyphenyl methacrylate **VM *3b*** (0.477 mg, 2.195 mmol), MMA (0.37 mL) and DMSO (0.99 mL) were placed under N_2 atmosphere. In a volumetric flask (10 mL), azobisisobutyronitrile (AIBN) (0.500 mg) were dissolved in degassed MMA. 0.19 mL of the AIBN solution were introduced in the reaction vessel and degassed again as before (MMA 0.523 g, 5.123 mmol in total; AIBN 9.61 mg, 0.0585 mmol, 0.8 mol%). The reaction mixture was then heated at 60 °C for 23 h. The resulting material was dissolved in $CHCl_3$ (20 mL) and then precipitated in MeOH (200 mL). The white fibers were filtered off on Büchner and washed repeatedly with MeOH. The solid was dissolved again with $CHCl_3$ and concentrated with a rotary evaporator. The residue was dried at 50 °C under vacuum, yielding 0.63 g (63%).

NMR Spectra

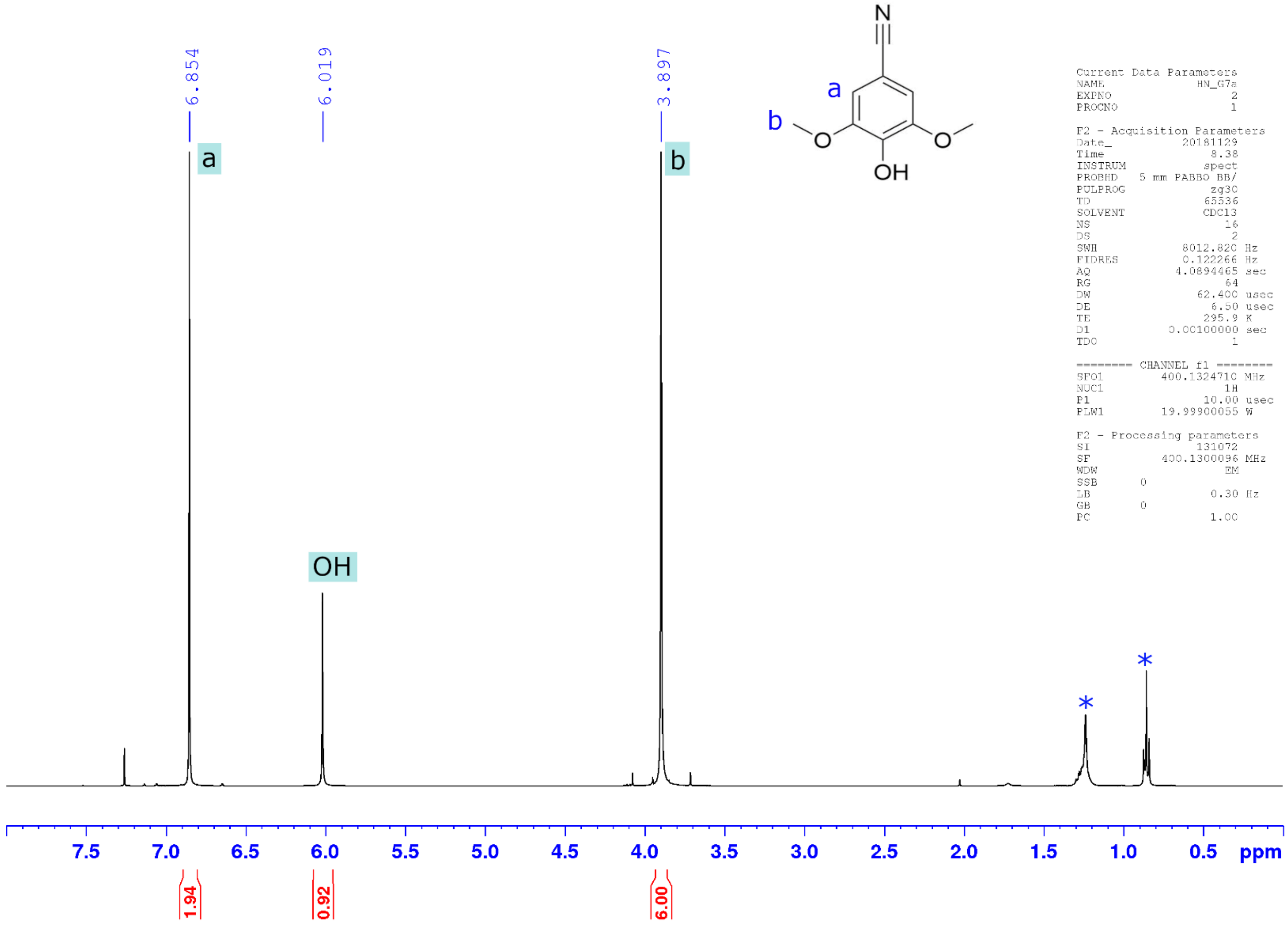

Figure S1. ^{1}H NMR spectrum of 4-hydroxy-3,5-dimethoxybenzonitrile (syringonitrile) ***2c*** (*indicates heptane signals).

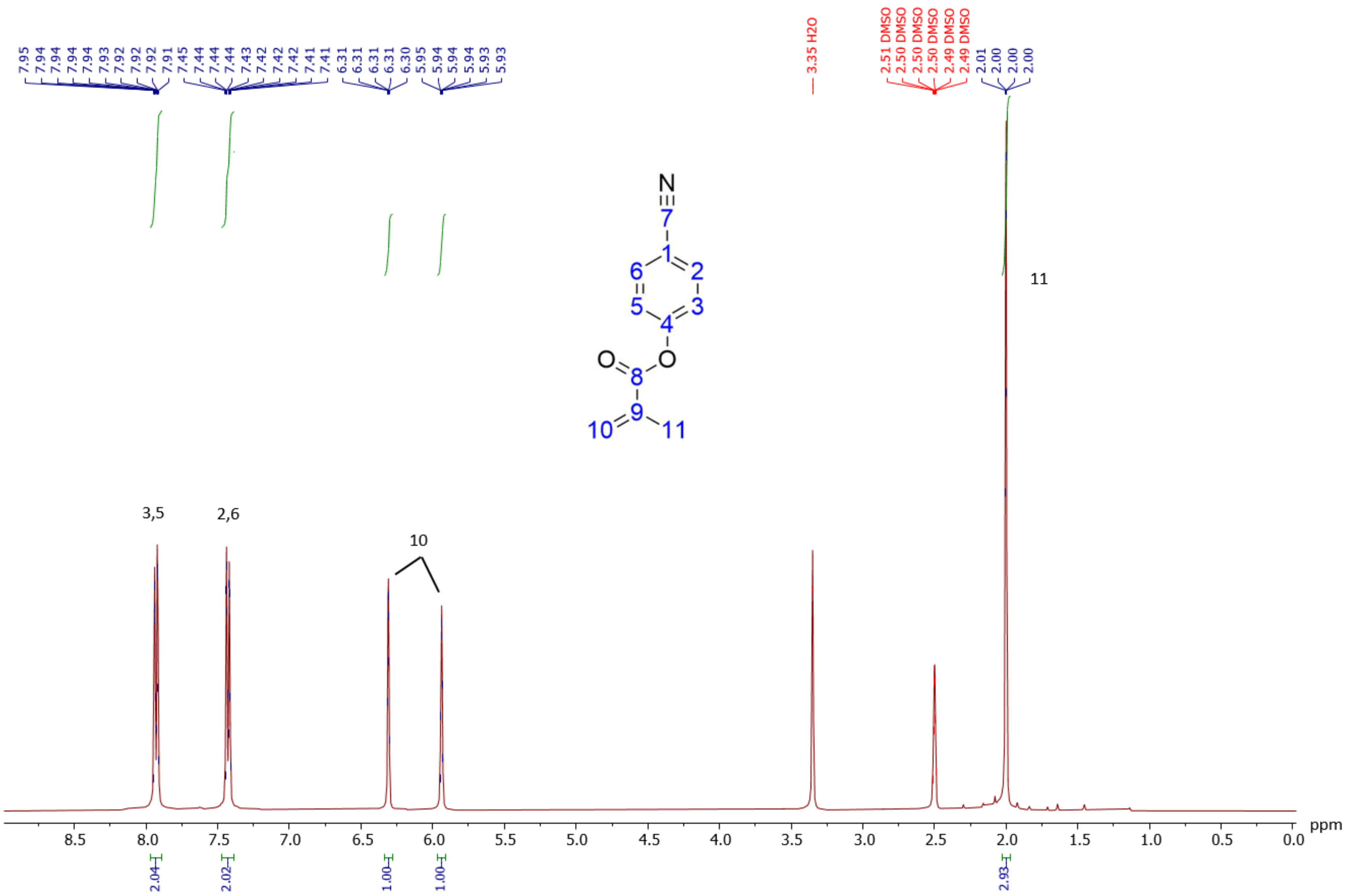

Figure S2. ^{1}H NMR spectrum of 4-cyanophenyl methacrylate (4-hydroxybenzonitrile methacrylate) **BM**.

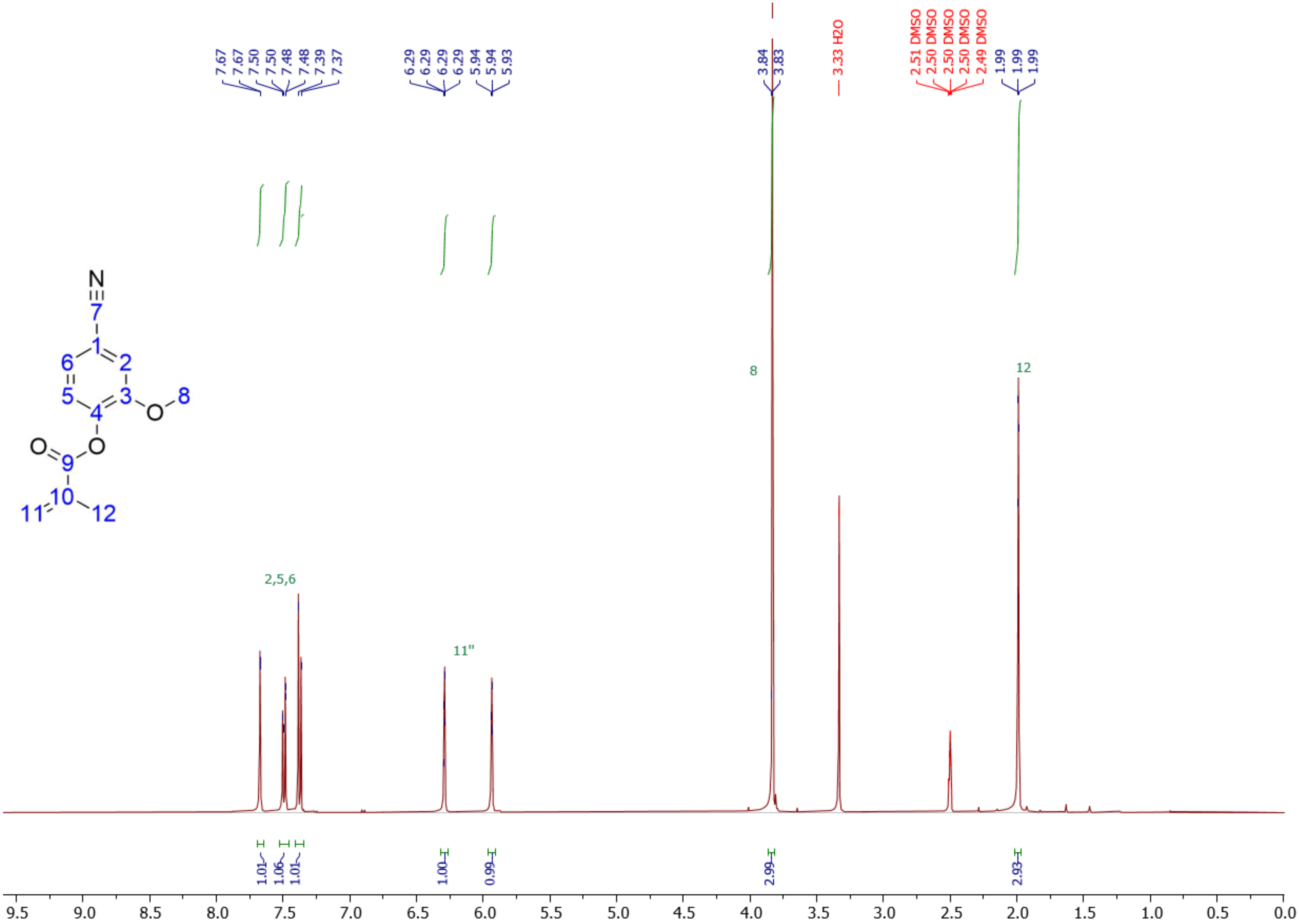

Figure S3. ^{1}H NMR spectrum of 4-cyano-2-methoxyphenyl methacrylate (vanillonitrile methacrylate) **VM**.

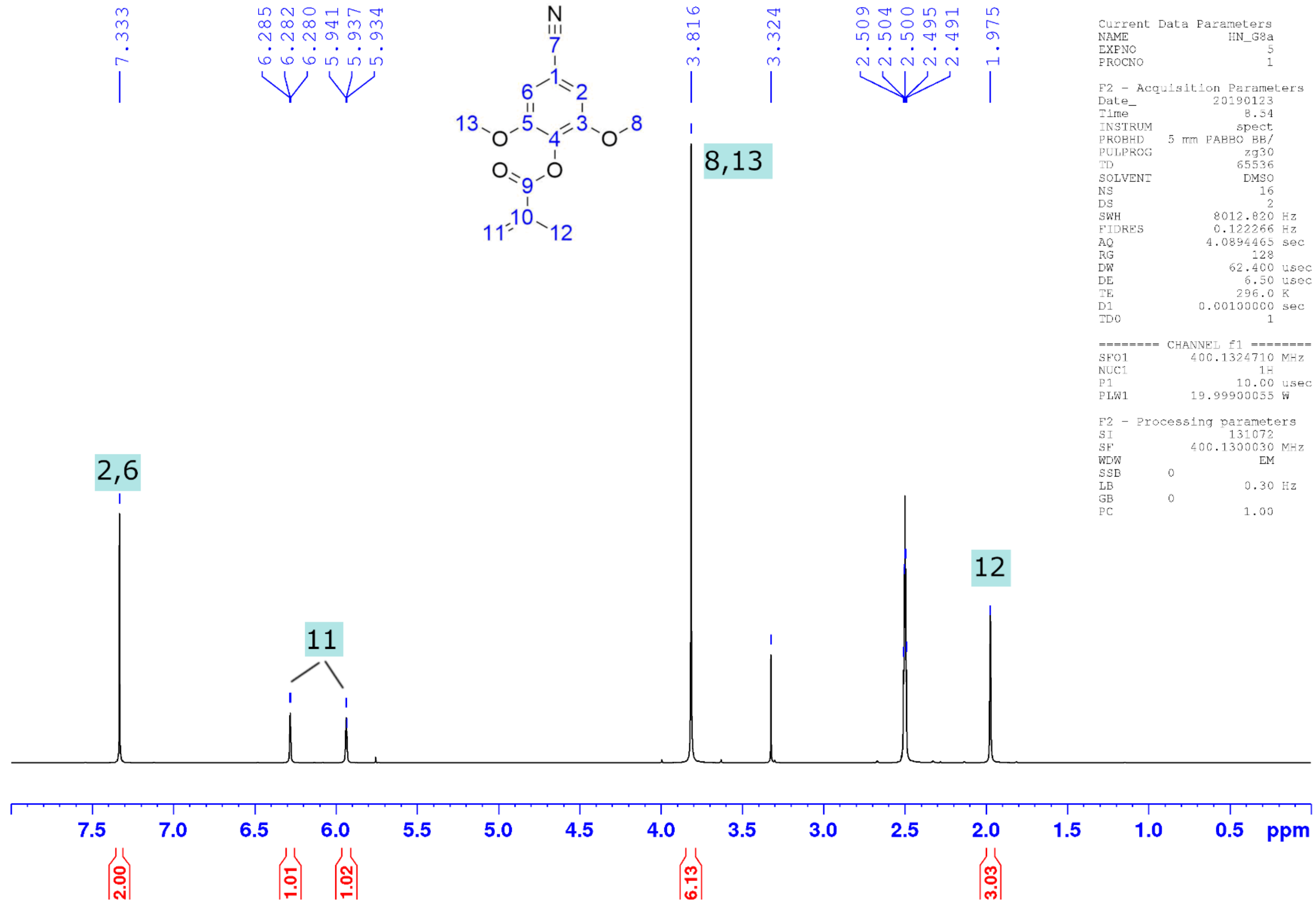

Figure S4. ^{1}H NMR spectrum of 4-cyano-2,5-dimethoxyphenyl methacrylate (syringonitrile methacrylate) **SM**.

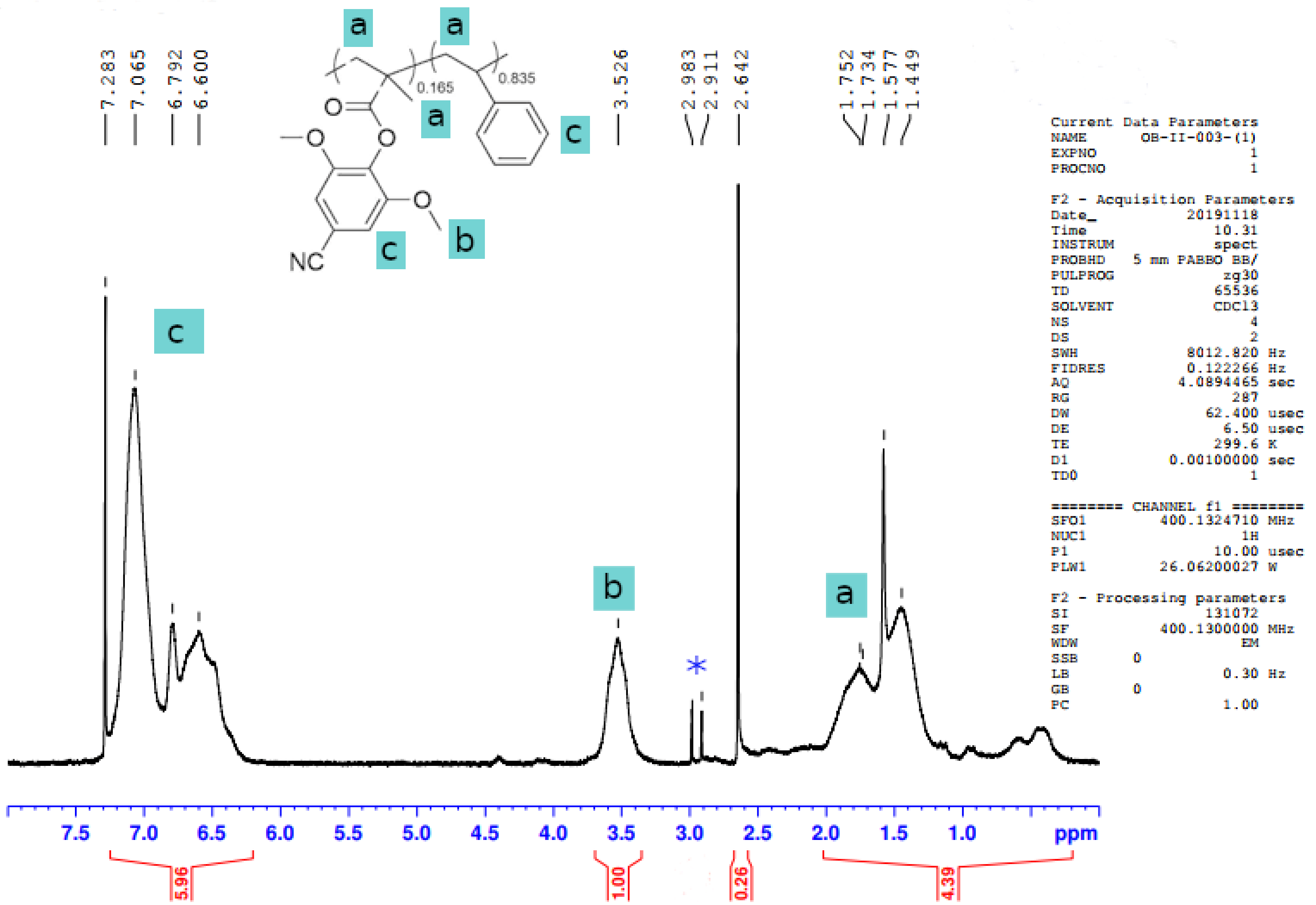

Figure S5. ^{1}H NMR spectrum of poly[styrene-*co*-(4-cyano-2,6-dimethoxyphenyl methacrylate)] **PSSM-16.** Signals **b** and **c** were used to determine the molar composition of the copolymers (*indicates DMF signals).

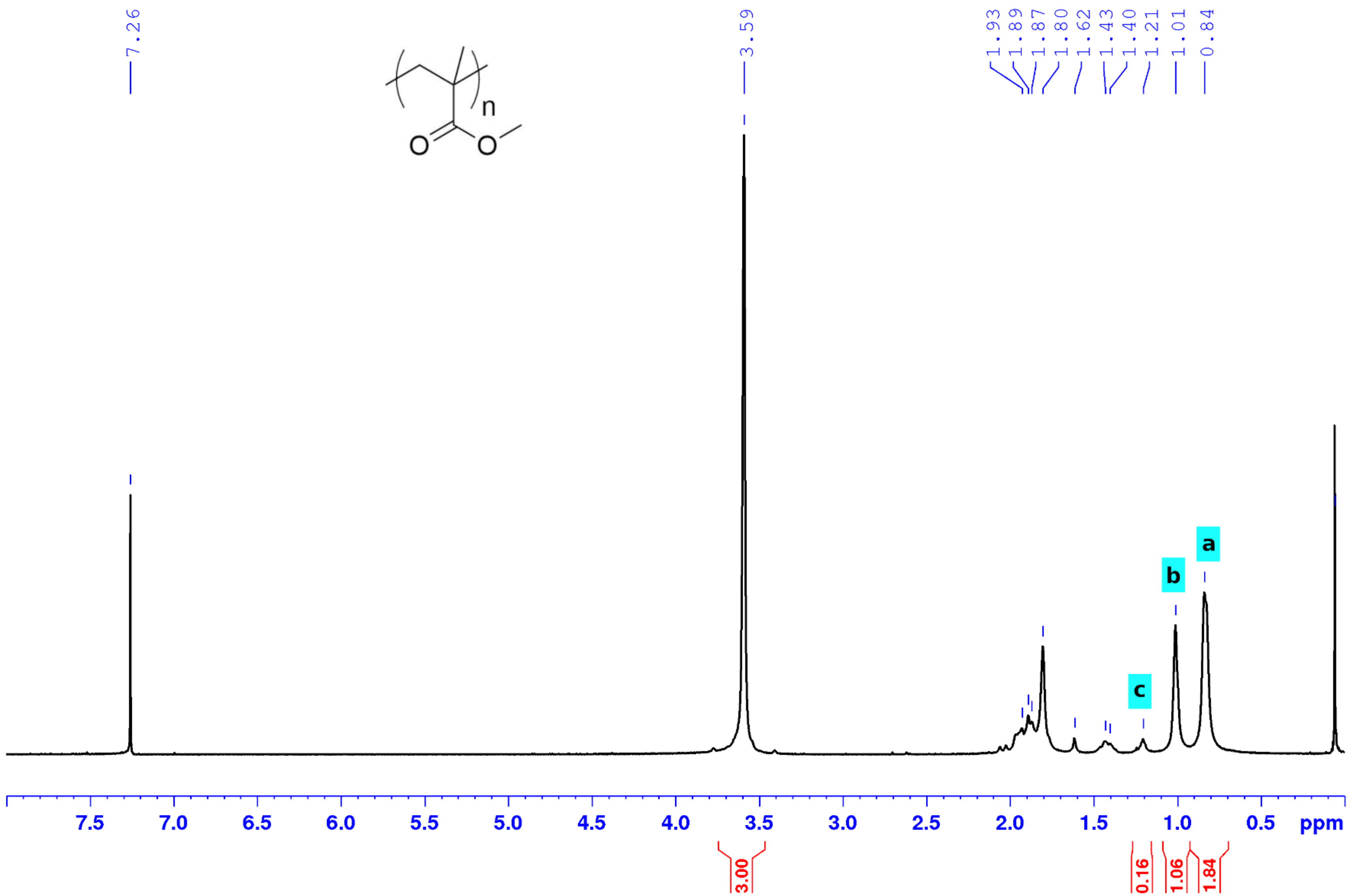

Figure S6. ^{1}H NMR spectrum of poly(methyl methacrylate) synthesized using the same procedure as the PMVM series Signals **a**, **b** and **c** were used to determine the *rr*, *rm* and *mm* triad fractions, respectively.

TGA Traces

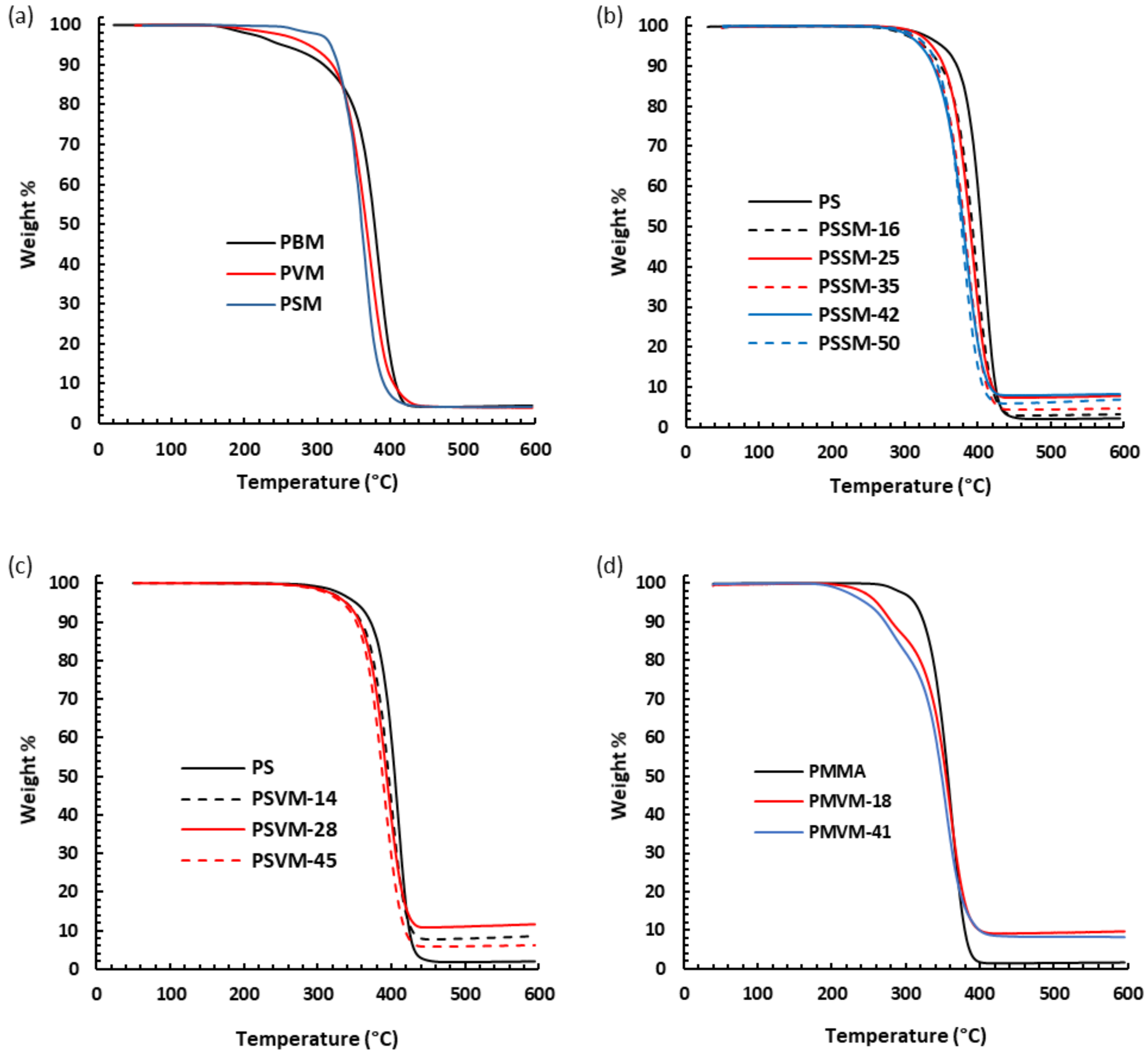

Figure S7. TGA traces of: (a) homopolymer PBM, PVM and PSM, (b) the PSSM series, (c) the PSVM series, and (d) the PMVM series under N_2 atmosphere at 10 °C min^{-1}.

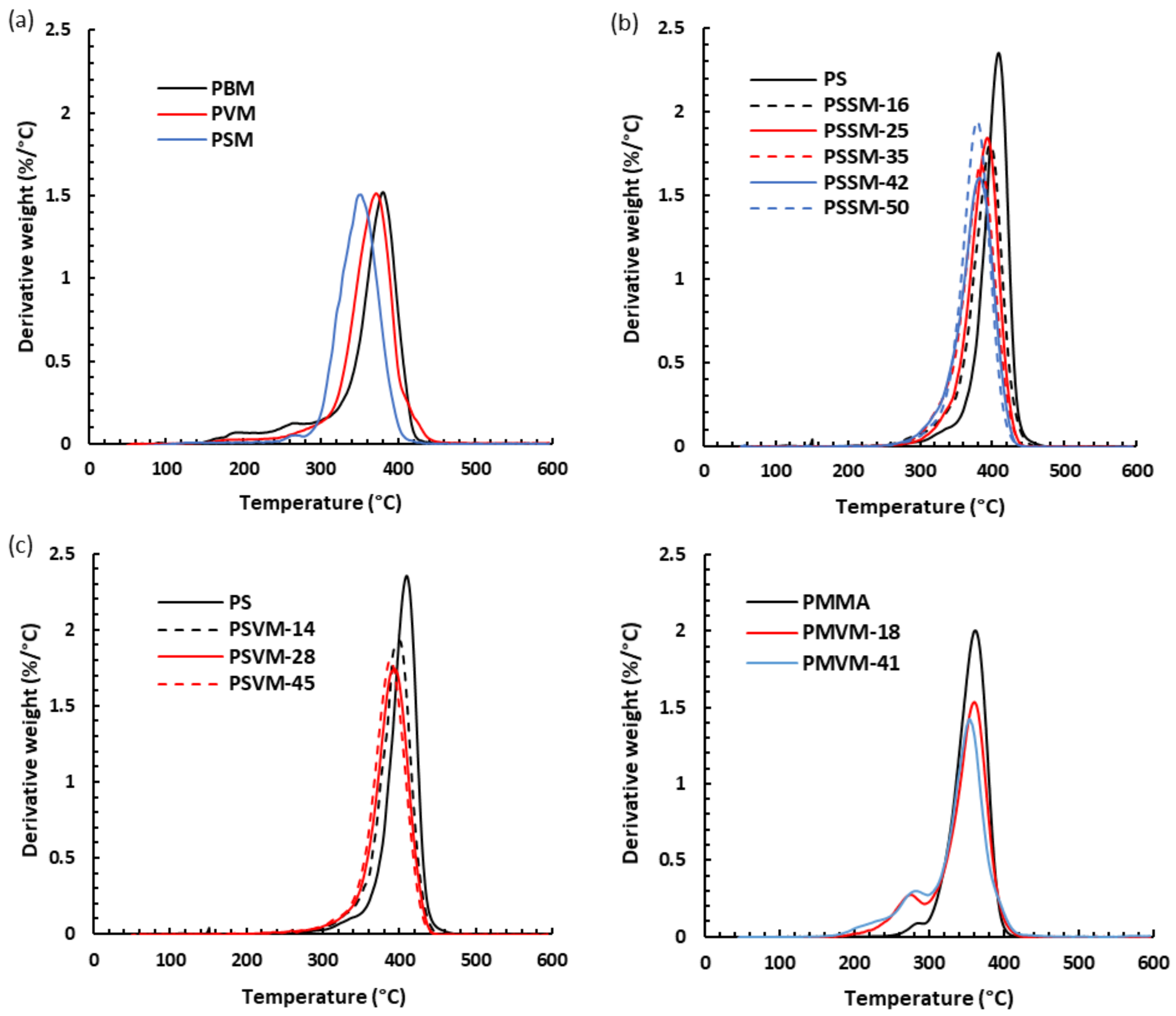

Figure S8. TGA derivative traces of: (a) homopolymer **PBM**, **PVM** and **PSM**, (b) the **PSSM** series, (c) the **PSVM** series, and (d) the **PMVM** series under N_2 atmosphere at 10 °C min^{-1}.

Flory-Fox plot

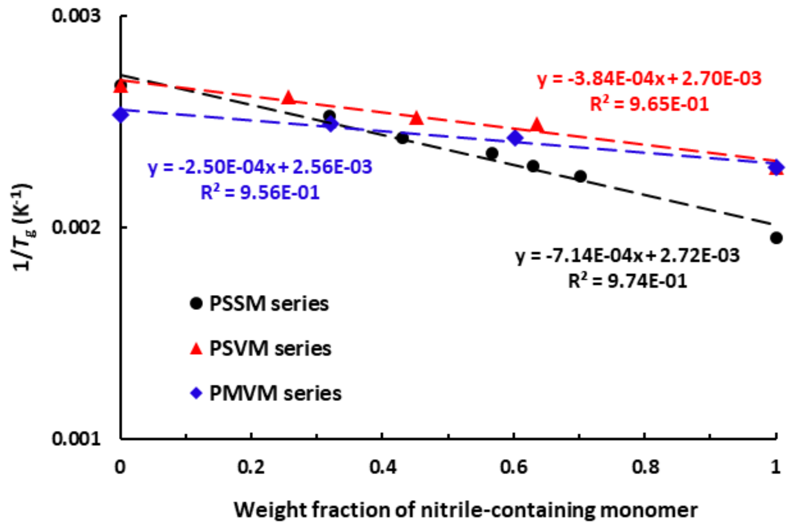

Figure S9. 1/T_g of the homopolymers and copolymers versus the weight fraction of nitrile-containing monomer. The fitted linear equations $y = ax + b$ are shown as dashed lines, where a = 1/T_g(1) – 1/T_g(2) and b = 1/T_g(2), with (1) referring to the nitrile containing polymer and (2) to PS or PMMA, respectively. The obtained parameters *a* and *b* for the three copolymer series are coherent with the theoretical values predicted using the Flory-Fox model.

SEC Curves (continued on next page).

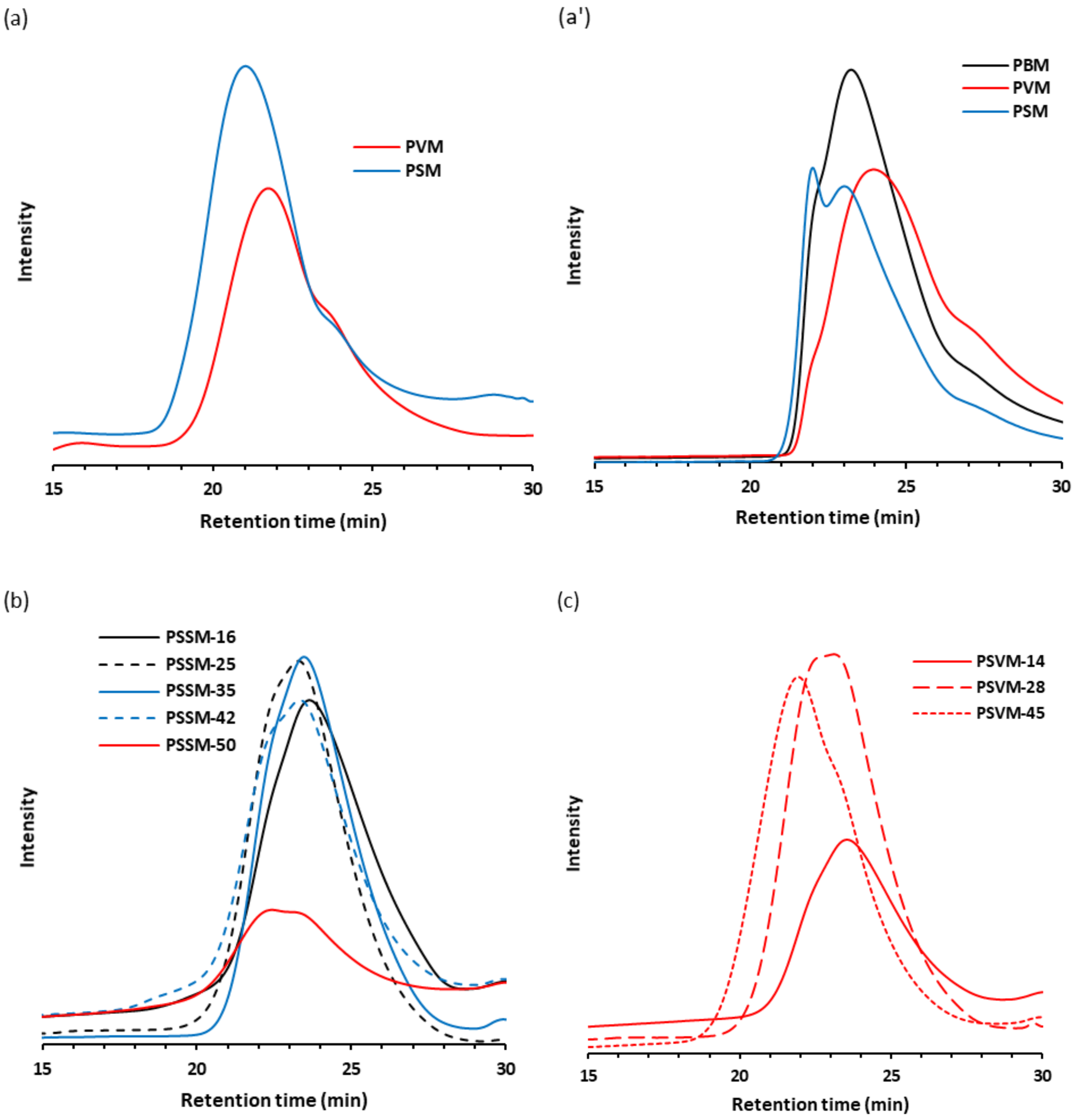

(d)

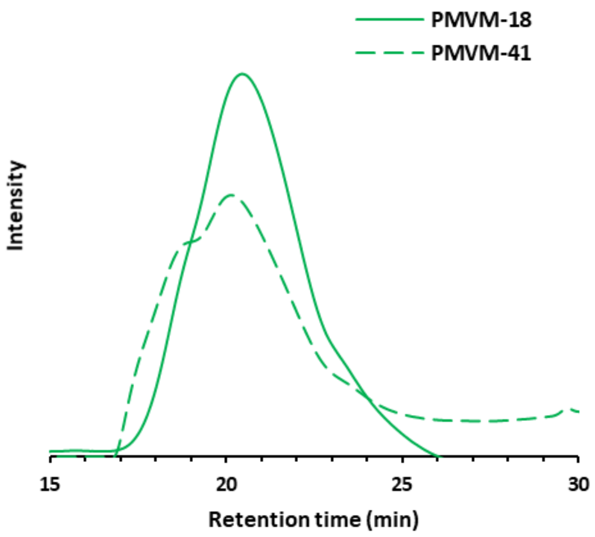

Figure S10. SEC of THF solutions of: (a) homopolymer **PVM** and **PSM**, (b) the **PSSM** series, (c) the **PSVM** series and (d) the **PMVM** series. "Intensity" is the differential refractive index (dRI) of the RI detector in arbitrary units. SEC in DMF of (a') homopolymers **PBM**, **PVM** and **PSM**.

Evaluation of the Solubility of Polybenzonitrile methacrylates

The solubility of the different polybenzonitrile methacrylates was investigated by mixing small samples (about 5 mg) with a range of selected solvents (1 mL). The mixture was stirred for 24 h at room temperature. The results of the dissolution tests were divided into two categories, soluble and insoluble, based on visual inspection. If the samples were found to be completely dissolved, they were considered as soluble; if not, they were considered as nonsoluble.

Table S1. Solubility of the nitrile-containing polymers at 21 °C.

	Solvent[a]								
**Polymer**	**H_2O** δ**=48 (s)**	**MeOH** δ**=43 (s)**	**1-BuOH** δ**=23 (s)**	**DMSO** δ**=25 (m)**	**THF** δ**=19 (m)**	**Et_2O** δ**=15 (m)**	**ACN** δ**=24 (p)**	**$CHCl_3$** δ**=19 (p)**	**Toluene** δ**=18 (p)**
**PS**	-	-	-	-	+	+	-	+	+
**PBM**	-	-	-	+	-	-	-	-	-
**PSVM-14**	-	-	-	-	+	-	-	+	+
**PSVM-28**	-	-	-	+	+	-	-	+	+
**PSVM-45**	-	-	-	+	+	-	-	+	-
**PVM**	-	-	-	+	+	-	-	-	-
**PSSM-16**	-	-	-	-	+	-	-	+	+
**PSSM-25**	-	-	-	+	+	-	-	+	-
**PSSM-35**	-	-	-	+	+	-	-	+	-
**PSSM-42**	-	-	-	+	+	-	-	+	-
**PSSM-50**	-	-	-	+	+	-	-	+	-
**PSM**	-	-	-	+	+	-	+	+	-
**PMMA**	-	-	-	+	+	-	+	+	+
**PMVM-18**	-	-	-	-	+	-	+	+	+
**PMVM-41**	-	-	-	-	+	-	-	+	+

[a]The symbols "+" and "-" indicate solubility and nonsolubility, respectively. Solubility parameters (δ, $MPa^{1/2}$) were obtained from the *Polymer Handbook*, (J. Brandrup, E. H. Immergut, E. A. Grulke, A. Abe, D. Bloch. *Polymer Handbook, 4th ed.*, John Wiley and Sons, New York, 1999), and the letters *s*, *m* and *p* denote strongly, moderately, and poorly hydrogen-bond-forming solvents, respectively.

Summary of previously reported T_gs of lignin-based polymethacrylates.

Table S2. T_gs of lignin-based methacrylate polymers reported in the literature (continued on next page).

Structure	Name of phenolic moiety	M_n (kg/mol)	T_g (°C)	Polymerization method	Ref.
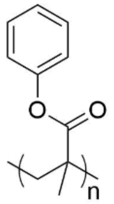	Phenol	41	119	RAFT	[53]
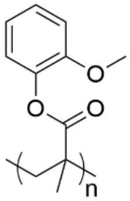	Guaiacol	38	115-120	RAFT	[25], [53]
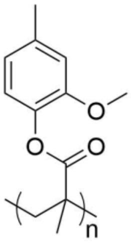	Creosol	34	121-132	RAFT	[25], [53]
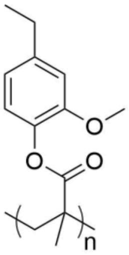	4-ethylguaiacol	35	108-116	RAFT	[25], [53]
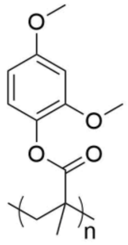	2,4-dimethoxyphenol	23	111	RAFT	[27]
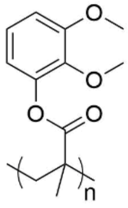	2,3-dimethoxyphenol	20	108	RAFT	[27]

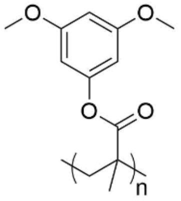	3,5-dimethoxyphenol	23	82	RAFT	[27]
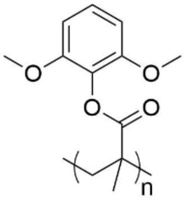	Syringol	24	203-205	RAFT	[26], [27], [53]
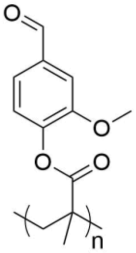	Vanillin	36	129-139	RAFT	[25], [53]
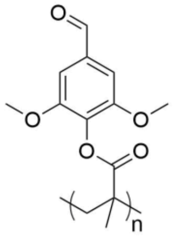	Syringaldehyde	29	201	RAFT	[53]
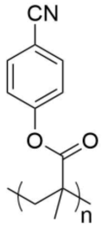	Benzonitrile	36	150	Free radical	Present study
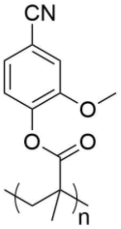	Vanillonitrile	23	164	Free radical	Present study
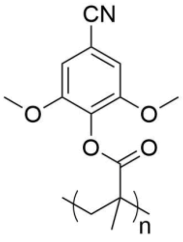	Syringonitrile	44	238	Free radical	Present study
